# Social robotics to support older people with dementia: a study protocol with Paro seal robot in an Italian Alzheimer’s day center

**DOI:** 10.3389/fpubh.2023.1141460

**Published:** 2023-06-21

**Authors:** Roberta Bevilacqua, Elvira Maranesi, Elisa Felici, Arianna Margaritini, Giulio Amabili, Federico Barbarossa, Anna Rita Bonfigli, Giuseppe Pelliccioni, Lucia Paciaroni

**Affiliations:** ^1^Scientific Direction, IRCCS INRCA, Ancona, Italy; ^2^Neurology Unit, IRCCS INRCA, Ancona, Italy

**Keywords:** older people, social robotics, dementia, psycho-social intervention, study protocol, Alzheimer’s disease, technology acceptability

## Abstract

**Introduction:**

The aging of the population and the high incidence of those over 80 lead to an inevitable increase in chronic degenerative diseases, such as dementia, resulting in increased morbidity and disability. Treatment of people with dementia involves both pharmacological and non-pharmacological interventions. In particular, robot-assisted therapy is a potentially useful treatment for dementia as it has the advantage of improving mood, encouraging social interaction and communication. The overall objective of the study is to evaluate the improvement in patient-perceived quality of life following the use of the Paro robot integrated with usual care in the older people with dementia.

**Methods and analysis:**

For this study, 20 patients with dementia are recruited and divided into Experimental Group (EG) and Control Group (CG). Twenty-four session of intervention are conducted, divided into 2 sessions per week, for 12 weeks. The therapy sessions last 20 min. The Experimental Group will receive a social robotic intervention with Paro combined with usual care; the Control Group will receive only the traditional therapy, consisting of cognitive stimulation (reality orientation therapy, cognitive training) and occupational activities (painting workshops, cooking workshops, garden therapy, music therapy, etc.). Paro is a seal-shaped robot designed to have a calming effect and elicit emotional responses in patients in hospitals, nursing homes, and retirement homes. Assessment will be performed at the baseline, at the end of intervention and 3 months after the end of intervention. During these phases, several scales will be administered to the patients, such as Quality of Life—Alzheimer’s Disease, Addenbrooke’s Cognitive Examination, the Rating Anxiety In Dementia scale and the Cornell Scale for Depression in Dementia, Quebec User Evaluation of Satisfaction, Neuropsychiatric Inventory, the Technology Acceptance Model.

**Discussions:**

The final goals of the present study are to evaluate the improvement in patient-perceived quality of life following the use of the Paro robot integrated with usual care in the older people with dementia.

**Ethics and dissemination:**

The study was approved by the Ethic Committee of the Istituto Nazionale Ricovero e Cura Anziani, Istituto di Ricovero e Cura a Carattere Scientifico (IRCCS INRCA) during the session of 12 April 2022. It was recorded in ClinicalTrials.gov on 23 November 2022 on the number NCT05626205. The study findings will be used for publication in peer-reviewed scientific journals and presentations in scientific meetings.

## 1. Introduction

According to data from the 2019 World Population Prospects, the planet’s population is aging: for the first time in history, in 2018 the “over 65 s” globally outnumbered children under the age of 5. In many countries around the world, improved general conditions, progress and socioeconomic well-being have contributed to a steady increase in the older population (1). In Italy, too, a significant demographic change is taking place. ISTAT (Istituto nazionale di statistica) data as of January 1, 2019 reports that the over-65 s number 13.8 million and represent 22.8 percent of the total population (2). The aging of the population and the high incidence of those over 80 lead to an inevitable increase in chronic degenerative diseases, such as dementia, resulting in increased morbidity and disability. Dementia has been defined as a global public health priority according to the World Health Organization and Alzheimer’s Disease International (2016) Report: “in 2010, 35.6 million people were reported to have dementia with estimates of a threefold increase by 2050, with 7.7 million new cases per year (1 every 4 s) and with an average survival, after diagnosis, of 4–8-years” (3). Dementia is the medical term used to refer to a group of degenerative diseases of the brain, usually arising in old age (but not exclusive to the older people), which result in a progressive decline in a person’s cognitive faculties such as memory, language, attention, movement, and the ability to plan and organize. Brain function is impaired to the point of interfering with a person’s normal social and working life. The main feature of dementia is the inability to perform everyday activities as a result of the deterioration of cognitive faculty.

Treatment of people with dementia involves both pharmacological and nonpharmacological interventions, that are recommended as adjunctive treatment.

In recent years, as technology has advanced, robots have been developed to assist the older population, particularly companion robots (4, 5). Two reviews, suggest that robot-assisted therapy is a potentially useful treatment for dementia as it has the advantage of improving mood, encouraging social interaction and communication, assisting individuals with their daily living, improving the well-being of the older people, and decreasing the workload of their caregivers (6, 7). Pet-robot interventions are effective treatment strategies for older people with cognitive impairment and dementia. In addition, these type of patient involvements have been shown to have positive effects on behavioral and psychological symptoms of dementia (8–11).

Social robots have also been used as substitutes for animals in therapy for people with dementia (12). Robots need less space, time or care. Their sensors can respond to environmental changes (movements, sounds…) by simulating interaction with the patient. They can monitor patients or be used in therapy.

Thus, after a review of the literature, among our research objectives is to evaluate the effects of using the newly acquired robotics at our institution (Paro), in patients attending the Alzheimer’s day care center. In fact, numerous studies have indicated that the utilization of Paro has the ability to enhance social interaction among residents, reduce stress and loneliness, and even boost immune system function (13, 14). Additionally, Paro has the potential to enhance both psychological and physiological well-being, and improve overall quality of life (15). These research findings strongly suggest that Paro can offer significant health benefits for older adult(s) individuals. Animal-assisted therapy (16), which integrates animals into human services, health, and education for therapeutic purposes, provides further evidence of the positive impact that Paro could have on the health of older adults. Most previous study tended to investigate the role of Paro in integrated environment including hospitals, homes, long-term care homes. In general, the effects of Paro on older adults in varying aged care facilities require further exploration. Moreover, the use of Paro robotic seal as an intervention has been shown to have a positive impact on various aspects of human well-being. Studies have demonstrated that interacting with Paro can lead to improvements in quality of life, cognitive functioning, and reduction in anxiety and depression. Furthermore, physiological measures of stress have shown a decrease during and after interaction with Paro. The use of Paro as an intervention has also been assessed in the context of job quality, with positive results in terms of job satisfaction and reduced stress levels for those working with older adult(s) or disabled individuals. Overall, the relationship between the intervention with Paro robotic seal and various aspects of human well-being is a promising area of research, with potential for further exploration and implementation in various settings. In particular, PARO has been found to improve social engagement in individuals with dementia, increased activity participation, and promote more spontaneous communication. Moreover, improvements to positive emotions and behaviors in individuals with dementia interacting with PARO has been demonstrated (17–19). PARO has been noted to help individuals become more active, smiling, relaxed and comfortable. In the light of these evidences, our purpose is to investigate, whether the robot can reduce some typical behaviors of the person with dementia (agitation), improve mood and cognitive status, stimulate social interaction, communication, and also reduce the caregiver burden of qualified care-related personnel, and last but not least, the acceptance of technology, compared to traditional treatment in a day care center.

The overall objective of the study is to evaluate the improvement in patient-perceived quality of life following the use of the Paro robot integrated with usual care in the older people with dementia.

## 2. Methods and analysis

### 2.1. Trial design

The study is designed as a pilot single blinded (outcome assessors) randomized controlled trial, with 3-month follow-up, in a group of 20 subjects with dementia, in order to evaluate the improvement in patient-perceived quality of life following the use of the Paro robot integrated with traditional intervention in the older people with dementia. The Experimental Group (EG) will receive a social robotic intervention with Paro combined with traditional one. The Control Group (CG), on the other hand, will receive only the usual care. Assessment will be performed at the baseline (T0), at the end of intervention (after 12 weeks -T1) and 3 months after the end of intervention (follow-up T2).

The primary aim is to evaluate the patient-perceived improvement in terms of quality of life through Quality of Life—Alzheimer’s Disease (QoL-AD), following the use of the Paro robot, integrated with usual care carried out within the IRCCS INRCA Alzheimer’s Day Center.

The Secondary aims are the evaluation of the improvement in cognitive status of the older person with dementia, in terms of orientation, attentional-executive, visuospatial functions, detected through the Addenbrooke’s Cognitive Examination (ACE-R); the assessment of mood improvement in terms of reduction of anxiety and depression, detected through the Rating Anxiety In Dementia (RAID) scale and the Cornell Scale for Depression in Dementia (CSDD); the assessment of the older person’s acceptance of the technology through Quebec User Evaluation of Satisfaction (QUEST 2.0), semi-structured interview and analysis of physiological activation during interaction, with the Noldus Face Reader system and direct observation by the operator; the analysis of behavioral symptomatology through Neuropsychiatric Inventory (NPI); the assessment of practitioner’s acceptance of technology through Technology Acceptance Model (TAM) and the assessment of job quality through semi-structured interview.

### 2.2. Study setting

The study is conducted at the Alzheimer’s Day Center of the IRCCS INRCA, Ancona, Italy. The last version (second version) of the current protocol is dated on 20th April 2022.

### 2.3. Participants

#### 2.3.1. Patient characteristics

The inclusion criteria are:

- Diagnosis of mild–moderate dementia according to the Diagnostic and Statistical Manual of Mental Disorders (DSM V).
- Mini Mental State Examination MMSE between 10 and 24.
- Signature of informed consent.
- Age 65 years or older.
- Have been placed in the Alzheimer’s Day Care Center for at least 3 months.
- Attending the Alzheimer’s Day Center for at least 6 months.
- Presence of a caregiver.

The exclusion criteria are:

- Sensory disabilities (visual and auditory).
- Difficulties in comprehension.
- Failure to meet inclusion criteria.
- Concurrent participation in other studies.
- History of syncopal episodes, epilepsy, and dizziness not controlled pharmacologically.
- Severe autonomic system dysfunction.
- Severe behavioral syndromes not compensated by medication.
- Lack of written informed consent.
- Active implanted and non-implanted medical devices.

#### 2.3.2. Operator characteristics

The inclusion criteria are:

- Alzheimer’s Day Center operators (Psychologists, Educators, Social and Health Care Workers with experience within the Alzheimer’s Day Center).

The exclusion criteria are:

- Concurrent participation in other studies.
- Lack of written informed consent.

### 2.4. Sample size

QoL-AD (20) was used to perform a sample size calculation. Assuming an effect size of 35%, the total sample size needed to capture this effect size was estimated to be 16 subjects, assuming a statistical power of 80%, a significance level of 0.05, two groups and 3 repeated assessments (one baseline, 2 follow-ups) in a within-between interactions ANOVA model. Even assuming a drop-out rate of 20%, the total required sample size would be 20 subjects (10 in each arm).

This sample size is assumed to be more than sufficient to capture variation even for secondary outcomes for which treatment effect sizes are assumed to be similar or larger than those identified for the primary outcome.

### 2.5. Recruitment

Patients are selected by the Alzheimer’s Day Center team of the Neurology Operating Unit, IRCCS INRCA, in the Ancona branch. These patients will be contacted by the center manager to schedule an interview with family members. Once they have verified that they meet the inclusion and exclusion criteria of the study and acquired the informed consent in duplicate, signed by both the patient and the family member, the center’s qualified staff will proceed to the baseline assessment with the questionnaires and clinical tests stipulated in the study design. A randomization technique based on a single sequence of random assignments is used. A list of random numbers generated by the computer is used and subject is assigned a number based on their order of inclusion in the study. According to this technique, the subjects are randomly assigned to one of the two study groups. At the end of intervention and after an additional 3 months, the above patients will be evaluated again.

### 2.6. Intervention

For this study, 20 patients with dementia are enrolled. The study design includes a recruitment phase (R), an initial evaluation phase before the start of treatment (T0), an evaluation at the end of treatment (T1 = 12 weeks from T0), and a follow-up evaluation (T2 = 24 weeks from T0). The Paro use sessions will be 20 min long, including 5 min of introduction to the robot and 15 min of actual use. The intervention will consist of 24 sessions, divided into 2 sessions per week, for the total duration of 12 weeks. The intervention will be considered valid, if individual participants have completed at least 80% of the sessions. Recovery of 2 sessions will be possible. During the treatment, video-recording of the interaction between the patient and Paro is provided, under the supervision of the practitioner.

#### 2.6.1. Technological intervention with Paro

The EG will receive intervention with the PARO robotic animal twice a week. Each session will last 20 min and will be continuous for three months. The meetings will take place in a separate, quiet room. Each session will consist of: (1) welcome of the participant, sanitization of the patient’s hands, presentation of Paro and preparation of the experimental setting involving video-recording of the interaction through camera placed in front of the patient who will be seated without a mask (5 min); (2) free use of Paro by the user (15 min). During the sessions, patients will be asked to dry their hands before interaction with Paro, and instructions will be given on how to avoid mustache contact with the user’s eyes. Throughout the interaction, the operator will maintain a distance of at least 2 m from the patient and wear an FFP2 mask. In addition, the operator will be asked to complete the observer’s checklist (See data collection sheet). Should the patient refuse to interact with Paro, the operator will encourage its use a maximum of 3 times before considering the session void.

The order of sessions, not to exceed 2 sessions per week, will be decided by the practitioner, including the possibility of offering Paro even if the patient manifests agitation or restlessness at any time of the day. Agitation, as assessed by the practitioner, may include verbal aggression, oppositional attitudes, and unrestrained movements. As studies (21–27) have shown, interaction with Paro contributes to mood improvement by helping the caregiver manage restless behavior.

#### 2.6.2. Traditional intervention

The CG involves the activities usually offered at the Day Care Center, such as cognitive stimulation (reality orientation therapy, cognitive training) and occupational activities (painting workshops, cooking workshops, garden therapy, music therapy, etc.).

#### 2.6.3. Equipment

Paro is a seal-shaped robot designed to have a calming effect and elicit emotional responses in patients in hospitals, nursing homes, and retirement homes. The robotic pup is 55 centimeters long, weighs less than three kilograms and is capable of moving its eyes, head, front and lower flippers. Paro also has many sensors that make him photosensitive and able to respond to tactile stimulation, even on his whiskers. Thanks to the presence of the three microphones, it is able to recognize the patient’s voice and is even capable of learning new information such as the subject’s name and behavioral characteristics, all thanks to advanced artificial intelligence. The robot also has the ability to “play” with the patient, thanks to autonomous behaviors that mimic those of a real pet.

### 2.7. Outcomes

All outcome measures follow a standardized operating procedure. Table 1 shows the primary outcome and the secondary outcomes.

**Table 1:** Outcomes and clinical assessments.

Outcome(s)	Clinical assessment
*Primary*: Quality of life (patient)	QoL-AD
*Secondary*: Cognitive status (patient)	ACE-R
*Secondary*: Anxiety (patient)	RAID
*Secondary*: Depression (patient)	CSDD
*Secondary*: Acceptance of technology (patient)	QUEST 2.0 semi-structured interview observation by the operator
*Secondary*: Behavioral symptomatology (patient)	NPI
*Secondary*: Acceptance of technology (operator)	TAM
*Secondary*: Quality of work	Semi-structured interview

QoL-AD, Quality of Life – Alzheimer’s Disease; ACE-R, Addenbrooke Cognitive Examination; RAID, Rating Anxiety In Dementia; CSDD, Cornell Scale for Depression in Dementia; QUEST 2.0, Quebec User Evaluation of Satisfaction; NPI, Neuropsychiatric Inventory; TAM, Technology Acceptance Model.

A summary of all data collected and when these are collected is provided ion Table 2.

**Table 2:** Schedule of assessment and outcome measures.

Scale	R	T0	T1	T2
Socio-demographics checklist	X			
Mini Mental State Examination (MMSE)	X			
Addenbrooke Cognitive Examination (ACE-R)		X	X	X
Hamilton Anxiety Rating Scale (HAM-A)		X	X	X
Cornell Scale for Depression in Dementia (CSDD)		X	X	X
Quebec User Evaluation of Satisfaction (QUEST 2.0)			X	
Semi-structured interview for patient technology acceptance			X	
Quality of Life – Alzheimer’s Disease (QoL-AD)		X	X	X
Neuropsychiatric Inventory (NPI)		X	X	X
Technology Acceptance Model (TAM)			X	
Semi-structured interview for technology acceptance and workload for operator			X	

R, Recruitment; T0 = Start of intervention; T1 = End of intervention (12 weeks from T0); T2 = Follow-up at 3 months (24 weeks from T0).

The scales used during the evaluations are described below.

#### 2.7.1. Mini-mental state examination

The instrument provides a score indicative of the degree and presence of any global impairment of cognitive function. Proposed for screening procedures. The score ranges from 0 to 30, scores ≥24 indicate normality, mild cognitive impairment between 18 and 23, between 11 and 17 medium cognitive impairment; ≤10 severe cognitive impairments. The reported score is adjusted for age and schooling (28).

#### 2.7.2. Addenbrooke cognitive examination revised

The ACE-R is a scale used in dementia. It consists of five different cognitive domains, for which separate scores can be calculated: attention/orientation (18 points), memory (26 points), verbal fluency (14 points), language (26 points) and visuospatial skills (26 points). The sum of the scores of the individual domains is equal to l00 which is the maximum score of the test. The administration takes an average of 15 min (29, 30).

#### 2.7.3. Hamilton anxiety rating scale

The HAM-A was one of the first rating scales developed to measure the severity of anxiety symptoms, and today it is still widely used in both clinical and research settings. The scale consists of 14 items, each defined by a set of symptoms, measures of both psychic anxiety (mental agitation and psychological distress) and somatic anxiety (anxiety-related physical complaints). Although the HAM-A remains widely used as an outcome measure in clinical trials, it has been criticized for its sometimes poor ability to distinguish anxiolytic effects from antidepressant effects and somatic anxiety effects from secondary somatic effects. The HAM-A does not provide any set of standardized questions. Despite this, the reported levels of reliability of the scale appear to be acceptable. Each item is scored on a scale from 0 (not present) to 4 (severe), with a total score range of 0–56, where <17 indicates mild, 18–24 mild to moderate, and 25–30 moderate to severe (31).

#### 2.7.4. Cornell scale for depression in dementia

This is a scale specially designed for the assessment of depressive symptoms in patients with dementia. The Cornell Scale uses a standardized set of items that are collected through an interview with a person who knows the patient (family member or caregiver) and semi-structured interview with the patient. It is therefore an observational instrument, which therefore does not require direct patient response to standardized formulated questions. The scale consists of 19 items, with responses that are graded from 0 (symptom absent) to 2 (symptom severe). It is one of the few scales validated in populations of subjects with dementia, including moderate–severe (32).

#### 2.7.5. Quebec user evaluation of satisfaction

The scale measures a person’s satisfaction with the device used. It consists of eight questions related to satisfaction with respect to the device in use and four questions with respect to the services associated with its provision. The user answers each question with a score ranging from 1 (completely dissatisfied) to 5 (very satisfied) (33).

#### 2.7.6. Quality of life—Alzheimer’s disease

The scale assesses the quality of life in patients with Alzheimer’s disease. It consists of 13 items (physical health, energy, mood, living situation, memory, family, marriage, friends, self as a whole, ability to do chores, ability to do things for fun, money, and life as a whole) to which the user gives a score between 1 and 4 (poor, fair, good, or excellent) (34).

#### 2.7.7. Neuropsychiatric inventory

The scale was developed to assess the neuropsychiatric symptoms and psychopathology of patients with Alzheimer’s disease. The 12 symptoms examined are: hallucinations, delusions, agitation, depression/ dysphoria, anxiety, euphoria/exaltation, apathy/indifference, disinhibition, psychoemotional irritability/lability, aberrant or aphinal motor activity, sleep disturbances, and appetite and eating disorders. Three aspects are investigated for each neuropsychiatric symptom: frequency (1, rarely, to 4, very frequently); severity (1, mild, to 3, marked); caregiver emotional and psychological distress (1, none, to 5, severe) (35).

#### 2.7.8. Technology acceptance model

The purpose of the TAM is to determine what factors influence the acceptance of a given technology, namely perceived usefulness and perceived ease of use, which also influences perceived usefulness. These factors determine attitude toward use, which in turn influences behavioral intention to use, which can be interpreted as acceptance of the technology. The questionnaire consists of 9 items, with responses on a Likert scale from 1 (“strongly agree”) to 5 (“strongly disagree”) (36).

### 2.8. Data management

All personally identifiable information gathered during the study will be managed and stored in compliance with the General Data Protection Regulation (GDPR) of 2018. The principal investigator will have control over the use of the study data. All records and documentation linked to the trial will be kept in accordance with relevant regulatory stipulations, and access to data will be restricted to authorized trial personnel.

### 2.9. Data analysis

A Plan of Analyses will be defined on the basis of which the analyses themselves will then be conducted. The first step of the analysis will be exploratory in nature. Descriptive analysis of the sample will be conducted using classical uni and bivariate statistical analysis techniques. Significant differences between outcomes and exposures will be compared using the Chi Square test, the Fisher Exact (in the case of categorical variables) or the *T*-test or the Anova test (in the case of comparisons of continuous variables between groups depending on whether or not they are normally distributed). In a second step, follow-up data analysis will be conducted in order to evaluate the effectiveness of the intervention. This phase of analysis will involve the use of the confounder assessment, that involves identifying and measuring potential confounding variables to ensure accurate estimation of causal effects, will be used. Techniques such as matching, stratification, or statistical adjustment through regression models will be used to account for confounding and obtain unbiased causal estimates. To assess the intervention’s effectiveness, techniques such as *t*-tests, chi-square tests, or analysis of variance (ANOVA) for continuous, categorical, or count outcomes, respectively will be used. These models compare the outcomes between the intervention and control groups, providing insights into the intervention’s impact. For the behavioral analysis, videos acquired during the interaction with Paro have been analyzed by the FaceReader software investigating facial expressions and generating a detailed report on the detected emotions, such as happiness, sadness, anger and fear. The information obtained from this analysis will be used to determine which emotion is most present during the interaction with Paro. Moreover, in the case of a semi-structured interview, data will be analyzed manually with the identification of themes, patterns, and commonalities across responses to provide insight into the research question.

## 3. Discussion

The aim of the present study is to evaluate an intervention protocol for patients with dementia designed to improve primarily perceived quality of life and secondarily cognitive ability, mood, behavioral symptomatology, and the older person’s acceptance of technology. In addition, the caregiver’s acceptance of technology and workload reduction will also be assessed. We focus on patients with dementia to test whether interaction with a companion robot, specifically an animal-like robot, combined with traditional cognitive training, can actually lead to benefits in terms of improved well-being and cognitive abilities.

The intervention involves robotic training with Paro combined with traditional 12-week training for 2 sessions per week lasting 20 min each. In each session, the older person will be able to freely interact with Paro, under the supervision of the operator, who will be responsible for observing that interaction. To study the effectiveness of the intervention, we will divide the population into an experimental group (EG), which will receive the combined training, and a control group (CG), which instead, will receive only the usual care. A possible limitation of the study should be the difficulty of generalizing the study involving patients at different state of the disease, in particular with a several dementia. This aspect could be solved by involving the informal caregiver in the study.

A strength of the study is the use of the Noldus Face Reader system for the analysis of physiological activation during interaction and the use of videotaped material that will be produced after each session, thanks to which even minimal facial expressions can be captured in the subjects. In addition, the choice of a one-on-one setting is appropriate for people with behavioral and psychological symptoms of dementia to see if there is improvement in this regard (37). Another relevant aspect of the protocol is the verification of the results obtained both at the end of the intervention and 3 months after the end, to note the maintenance and generalization of the results obtained.

## 4. Ethics and dissemination

### 4.1. Ethics and confidentiality

The research underwent approval by the Ethics Committee of the Istituto Nazionale Ricovero e Cura Anziani, Istituto di Ricovero e Cura a Carattere Scientifico (IRCCS INRCA) during the meeting held on April 12, 2022. In case of any modifications to the protocol, the same Ethics Committee will be notified. This committee also acts as the data monitoring committee, periodically evaluating the progress of the protocol and ensuring compliance with the principles of the Declaration of Helsinki and Good Clinical Practice guidelines. Written informed consent will be obtained from all participants involved in the study. All personal data collected during the trial will be managed and stored in accordance with the General Data Protection Regulation (GDPR) of 2018 (38). The principal investigator will have control over the usage of the study data. All data and documentation pertaining to the trial will be stored in accordance with relevant regulatory requirements, and access to data will be restricted to authorized trial personnel.

### 4.2. Dissemination of research findings

The study findings will be used for publication in peer-reviewed scientific journals and presentations in scientific meetings. Summaries of the results will also be made available to investigators for dissemination within their clinics.

## Data availability statement

The datasets generated, used and analyzed during the trial will be available from a specific data repository or from the corresponding author upon reasonable request.

## Ethics statement

The studies involving human participants were reviewed and approved by Ethic Committee of the Istituto Nazionale Ricovero e Cura Anziani, Istituto di Ricovero e Cura a Carattere Scientifico (IRCCS INRCA). The patients/participants provided their written informed consent to participate in this study.

## Author contributions

RB: study concept and design. EF and LP: acquisition of data. RB, EM, GA, and FB: analysis and interpretation of data. EM, RB, and AM: drafting of the manuscript. GP, AB, and LP: critical revision of the manuscript for important intellectual content. EM and RB: writing—review and editing. All authors contributed to the article and approved the submitted version.

## Conflict of interest

The authors declare that the research was conducted in the absence of any commercial or financial relationships that could be construed as a potential conflict of interest.

## Publisher’s note

All claims expressed in this article are solely those of the authors and do not necessarily represent those of their affiliated organizations, or those of the publisher, the editors and the reviewers. Any product that may be evaluated in this article, or claim that may be made by its manufacturer, is not guaranteed or endorsed by the publisher.
